# The Patient Engaged Research Center's Sustainable Funding Framework: A Path Towards Sustainable Patient Engagement in Care and Research Within a Health System

**DOI:** 10.1002/lrh2.70047

**Published:** 2025-10-30

**Authors:** Paige Coyne, Leah Copeland, Dana Murphy, Ashley Redding, Christine C. Johnson, Karen E. Kippen, Sara Santarossa

**Affiliations:** ^1^ Department of Public Health Sciences Henry Ford Health Detroit Michigan USA; ^2^ College of Human Medicine Michigan State University Lansing Michigan USA; ^3^ Henry Ford Health + Michigan State University Health Sciences Detroit Michigan USA

**Keywords:** patient participation, patient‐centered care, sustainable development

## Abstract

**Background:**

Despite growing acknowledgement that patient engagement (PE) in research, quality improvement, and clinical care is important, models showcasing how learning health systems (LHSs) can sustain long‐term PE across endeavors remain scant. Henry Ford Health's (HFH) Patient Engaged Research Center (PERC) provides a replicable example by which other LHSs can feasibly sustain/grow PE across research, quality improvement, and clinical care in a more efficient and cohesive manner.

**Methods:**

To support its current infrastructure, PERC obtains financial support from an array of sources, including internal health system funding, external grant funding, and philanthropic support. In addition, PERC has created a Sustainable Funding Framework (SFF) and offers à la carte patient‐centered services to further diversify its funding and ensure the sustainability of PE throughout the system. PERC utilizes a four‐step SFF to offer expertise in conducting patient‐centered research, as well as operational and programming support for PE‐related initiatives at HFH and within the broader community. The steps are as follows: awareness/need recognition, intake process (intake form, intake meeting, and invoice), project status (approval or not), and project details/start date. Example services include, but are not limited to, instrument development (surveys, moderator guides for interviews/focus groups), facilitation/transcription (surveys/interviews/focus groups), data analysis and reporting (mixed methods and qualitative), Patient Advisor recruitment and training, development/maintenance of Patient and Family Advisory Councils, placement of patient advisors on committees/councils/projects, and grant writing.

**Discussion:**

PE in research, quality improvement, and clinical care within most health systems is often siloed and disjointed, lacking a sustainable financial or work process model. PERC's SFF provides a promising and replicable example by which LHSs can feasibly sustain and grow PE across research, quality improvement, and clinical care delivery, as well as incorporate this data in a feedback loop to improve all three.

## 1 Introduction

### 1.1 Patient Engagement (PE)

PE is increasingly recognized as an integral component of health care and clinical research [1]. Health care systems, especially those who are already or wanting to become learning health systems [2, 3, 4], are beginning to acknowledge that patients and their families possess valuable information that can and should be used to improve care processes, systems, and policies, as well as for knowledge generation [2, 3, 5, 6, 7, 8]. Defined as the active involvement of patients and other stakeholders (e.g., families, their representatives, health professionals, researchers, community members) across all levels of the health care system (e.g., direct care, organizational design and governance, policy making) and research (e.g., planning, conduct, analysis, and dissemination) [9, 10], PE has the potential to enable health care systems to improve health outcomes, while also reducing costs [11, 12, 13, 14]. Among many positive outcomes, PE can improve quality of care, patient satisfaction, safety, and compliance (e.g., adherence to treatment recommendations), as well as patient self‐efficacy, resulting in reduced lengths of stays and emergency readmissions [6, 7, 8, 15]. Similarly, for research, PE can help with research prioritization, increase study enrollment rates, support funding securement, assist in study protocol design, and support the selection of relevant outcomes [6, 16, 17, 18].

### 1.2 Henry Ford Health (HFH) and the Patient‐Engaged Research Center (PERC)

In 2014, HFH sought to set a new standard of PE with the creation of its PERC. Housed within the Department of Public Health Sciences, PERC's mission is to *translate the patient voice into evidence‐based care through community engagement and world‐class research methods*. Initial funding for PERC was obtained through an Agency for Healthcare Research and Quality (AHRQ) non‐renewable R24 award, which was co‐developed and submitted by a diverse group of HFH researchers, as well as patients and other stakeholders. Using this funding, PERC began building patient engagement and patient‐centered outcomes research infrastructure and a Flexible Engagement Model (FEM) [19] to recruit, onboard, train, and retain Patient Advisors (PAs; i.e., patients, caregivers, and family members with healthcare experiences) to engage in quality improvement and research through its PA Program, which uses a seven‐step process (recruitment, informal screening interview, PA orientation, PA project assignment, PA liaison, retention, and dissemination), and includes the collection of demographic information (age, gender, race/ethnicity, address) and the creation of PA biographies.

Although PERCs infrastructure continues to evolve, at present, PERC maintains two arms: (1) Research Operations and (2) Program Operations. Currently (2025), the Research Operations Arm houses a Scientific Director and three other researchers (one at the doctoral level and two at the master's level) who spearhead and support a variety of patient‐centered research projects. The Program Operations Arm consists of a Program Manager (PM) and a Program Coordinator, whose responsibilities are to run the PA Program using the FEM [19]. PERC's decision to build out a patient‐centered outcomes research infrastructure and a FEM has proved successful in the 10 years since its inception, with the development and training of over 360 PAs who are currently serving on 55+ projects, committees, and councils across the health system to support patient engagement. PAs represent diverse geographical (*n* = 9 out of state, *n* = 4 out of country, i.e., Canada), racial (35% minority representation), and socioeconomic (over 180 different zip codes across 17 counties in Michigan) statuses to ensure varied patient perspectives are brought to the table.

### 1.3 PERC and Sustainability

Centers like PERC are often created through infrastructure grants. Once such funding expires, these centers typically become financially unsustainable and patient engagement across research, quality improvement, and clinical care dwindles. To continuously support its current infrastructure (staffing, computer software and equipment, supplies, etc.), which enables patient engagement in research, quality improvement, and clinical care, PERC obtains financial support from an array of sources, including internal learning health system funding, external grant funding, and philanthropic support [19]. In addition to the aforementioned funding sources, PERC recently created a Sustainable Funding Framework (SFF) and began offering à la carte patient‐centered services (e.g., placement of PAs on research projects, grant writing, instrument development, mixed methods and qualitative analysis) to further diversify its funding portfolio and generate additional revenue. In this article, we provide a detailed overview of PERC's SFF, a promising and replicable means by which learning health systems can offer patient‐centered services in order to sustain and grow PE across research, qualitative improvement, and clinical care delivery to generate data and disseminate knowledge.

## 2 Sustainable Funding Framework (SFF)

Various frameworks, guiding principles, and practical recommendations have been developed to support and promote PE. However, these resources are frequently designed with different objectives in mind and often times fail to address the invisible labor of PE. Some specifically target PE within the context of medicine development [20, 21, 22, 23, 24, 25, 26], whereas others address broader aspects of patient involvement in research or the healthcare system as a whole [5, 27]. This diversity of tools enables stakeholder organizations to select those most aligned with their specific requirements. Nonetheless, many of these frameworks tend to be temporary, with limited applicability across different settings, which can impede the establishment of a consistent, long‐term approach to embedding PE beyond isolated initiatives. Moreover, patient engagement is often treated superficially, emphasizing individual preferences and satisfaction rather than deeper integration.

**FIGURE 1:**
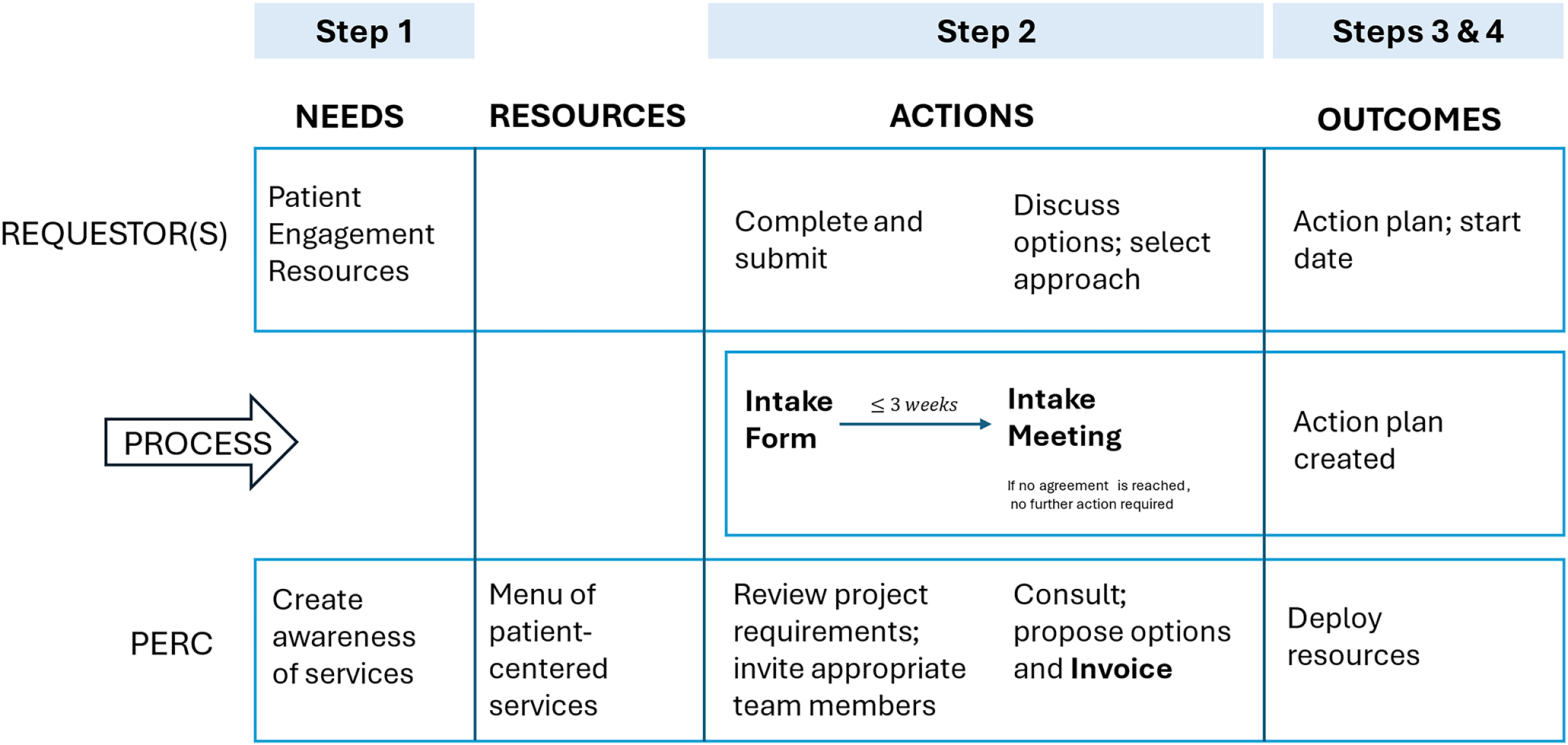
Patient Engaged Research Center's (PERC) Sustainable Funding Framework.

To address these challenges and promote a more structured and sustained approach to patient engagement, PERC implements a systematic four‐step framework designed to deliver patient‐centered services both at HFH and within the broader community (see Table 1 for a complete list of services). The steps are as follows: (1) awareness/need recognition, (2) intake process (intake form, intake meeting, and invoice), (3) project approval, (4) project details and start date. Each step is described in greater detail below (Figure 1). Examples are presented in Table 2.

**TABLE 1:** List of services offered by the Patient Engaged Research Center (PERC) through its Sustainable Funding Framework (SFF).

Service	Explanation
Instrument development	Development and/or review of surveys, moderator guides for interviews (one‐on‐one) and focus groups (multiple participants). Types: *structured* (predetermined questions in a set order, often closed ended, e.g., yes/no, multiple choice), *semi‐structured* (mix of structured and unstructured, where questions are often open ended and prepared in advance but allow for flexibility in order, phrasing, and use of prompts additional questioning), and *unstructured* (questions are not predetermined and no set pattern)
Facilitation	Following a moderator guide (can be created by Patient Engaged Research Center (PERC) as a separate service), conducting interviews and/or focus groups. Includes: *preparing the room* (physical or virtual), *opening the session* (introductions, explain ground rules, housekeeping reminders), *asking questions* (including giving space for all participants to speak), *taking notes/recording the session*, and *closing the session* (reflect on main ideas, ask for any last comments, thank individual or group for participating, reminder about compensation if applicable, and collecting notes/stopping and saving the recording)
Transcription	Converting audio from interviews and/or focus groups to text. Types: *edited* (edited for readability, conciseness, and clarity), *verbatim* (capture every sound made, including throat clearing and verbal pauses such as “um”), *intelligent verbatim* (edit out distracting fillers and repetitions)
Dissemination	Sharing of research study or quality improvement instruments (e.g., surveys) and other relevant or related information (e.g., recruitment flyer) to PERC's Patient Advisor Pool
Data analysis and reporting (mixed methods and qualitative)	Data from surveys, interviews, and/or focus groups are analyzed and reported back to requestor. Options for qualitative and mixed methods reporting are available. Types: *preliminary* (analysis of specific question or interviews/focus groups when research is still ongoing), *full analysis* (in‐depth report of all interview/focus group data), *post‐report* (general summary of data)
Patient recruitment and coordination	Identifying, attracting, enrolling, and scheduling/coordinating patients as participants for research studies, quality improvement projects, or other initiatives. Type: within PERC's Patient Advisor Pool, within the health system, beyond health system
Development and/or maintenance of Patient and Family Advisory Councils (PFACs; ~10–15 advisors) [19]	Works with research and quality improvement requestors to clarify the purpose of their PFAC and identify the person who will be responsible for facilitating all meetings (can be the requestor themselves or someone else of their choosing), known as the Patient Advisor Liaison (PAL). PERC then leads a series of planning meetings with the PAL to train them in facilitation and work through PFAC logistics (e.g., eligibility of Patient Advisor for PFAC, structure of meetings, frequency of meetings, and location of meetings, etc.). Next, PERC will work with the PAL to recruit and select Patient Advisors for the PFAC. PERC supports the PAL in running the PFAC for the first 3–6 months of meetings, including the creation of a group charter for members to abide by, developing meeting agendas, scheduling/coordinating meetings Afterwards, the PAL will run the council on their own. For the duration of a PFACs existence, PALs are able to contact PERC at any time for support and attend quarterly PAL meetings with the PERC team to discuss any challenges or successes their own PFAC is having.
Placement of Patient Advisor on committee or council	Identify Patient Advisors (usually 1–2) from PERC's Patient Advisor Pool to be placed on new or existing committees and councils within the health system. PERC works with committees and councils to select Patient Advisors who would be well suited, based on their experiences and expertise. In the rare instance that PERC's Patient Advisor Pool lacks a suitable Patient Advisor, PERC will ask its Patient Advisor Pool if they know of anyone who might be suited for the role and can take them through the onboarding process to become a member of the Patient Advisor Program.
Patient‐centered research design and grant writing support	Researchers and physician scientists from within the health system (and beyond) can request support from PERC in the design of future research studies to make them more patient‐centered via a collaborative process that includes in‐person/virtual meetings and review of study documents. Patient‐centered research studies are designed to increase collaborative efforts between researchers and the participants (patients). Engagement, knowledge exchange and dissemination, as well as action and reflection are key components of these types of designs. PERC can assist in all aspects of study design and execution. Examples of novel designs with which PERC has expertise include, but are not limited to: photovoice, body mapping, community‐academic partnerships, participatory action research, inclusive social media research, social network analysis.
Flyers/material development	Creation of patient‐centered informational materials, recruitment materials, flyers and other patient friendly graphic design.

**TABLE 2:** Use cases of Patient Engaged Research Center's (PERC) Sustainable Funding Framework (SFF).

Project	Step 1: Awareness/need recognition	Step 2 and 3: Intake process and project approval	Step 4: Project details and start date	Outcome	Connection to learning health systems
Intermittent Fasting in Gynecologic Cancer	**Requestor:** – Women's Health and Cancer Bench Scientist **Referral:** – Word of mouth from PHS Department Chair and Women's Health Research Collaborative	**Intake form** (December 2021): – Services requested: Focus Groups – Intake meeting members: Requestor to attend **Intake meeting** (December 2021): – Attendees: PERC's Scientific Director, Project Manager, and an Epidemiologist – Introduction and Overview of PERC – PERC's Project Manager provided overview of PERC and services offered with focus on qualitative research – Discussion about objectives and goals for project – Services needed: study design, Institutional Review Board (IRB) assistance, recruitment, REDCap survey and analysis, moderator guide development, focus group (conduct, transcribe, preliminary and full analysis) – Budget: discussed options that included percent full time equivalent (%FTE; e.g., 5% of time for 12 months) of staff member, flat rates, and combination **Invoice:** 2 budget options (one lower and one higher in cost) **Project approval** (January 2022): – Funding: Requestor had internal funding available – Invoice: Combination of flat fee and %FTE selected	**Project details:** – Deliverables: survey data and analysis, focus group transcripts, preliminary and full analysis – Invoice: flat fee for recruitment, IRB support, and distribution of participant incentives. Epidemiologist at 5% FTE – Timeline: 3 months **Start date:** May 2022	– Manuscript published in August 2023 in BMC Research Notes [28] – Conference presentation in November 2023 [28] **Lesson learned** – Working with interdisciplinary groups can be challenging, – The bench scientist leading the study had never worked with humans and was not familiar with the IRB process – Solution: PERC delayed the project timeline in order to support the bench scientist's submission submit to IRB – Lesson: build in extra time for IRB and ensure accurate budgeting of IRB services needed (i.e., clarify PERC tasks related to IRB submission)	– Interdisciplinary collaboration (i.e., bench scientists, research scientists, patients) to improve randomized controlled trial design – Engaging patients in research (patients involved in design of study) – Sharing findings with other learning health systems (e.g., manuscript publication and conference presentation)
Henry Ford Health—Detroit Patient and Family Advisory Council (PFAC)	**Requestors** – Director of Care Experience and Destination: Grand Steering Committee^a^ **Referral:** – Director of Care Experience was referred to PERC via word of mouth from Public Health Sciences' Department Chair	**Intake form** (September 19, 2023): – Service requested: PFAC development – Patient Advisor (PA) eligibility: received care at Detroit campus within last year – PA commitment: monthly meetings – Intake meeting members: Director of Care Experience and Care Experience Manager (Detroit) **Intake meeting** (September 28, 2023): – Attendees: PERC's Scientific Director, Project Manager, and Project Coordinator, as well as Director of Care Experience and Care Experience Manager (Detroit) – Identified Patient Advisor Liaisons (PALs): Director and Manager – PERC's Project Manager provided overview of PERC and services offered with focus on PFAC development – Discussion about objectives and goals for PFAC – Budget: Not applicable^b^ **Invoice:** Not applicable^b^ **Project approval** (September 2023): – Funding: Costs covered by PERC through internal funding – Invoice: Not applicable^b^	**Project details:** – Recruitment: October 2023 – Development of Strategic Plan: October–November 2023 – PERC supporting PALs and PFAC: 6+ months – Kick‐off meeting (1st PFAC meeting): December 2023 – Continued monthly meetings **Start date**: October 2023	**Provided feedback on the following initiatives to increase patient‐centeredness**: – Ask Me 3 [29] educational initiatives in emergency department – Advanced directives – Patient education materials regarding mobility – Destination: Grand^a^ **Lesson learned:** – PFAC members were eager to share their experiences and perceptions but struggled to identify when it is appropriate to do so, creating a tense environment – Solution: PERC worked with PFAC members to repeat training from FEM [19] as a reminder of how/when to share their feedback on various initiatives. The atmosphere improved significantly – Lesson: provide ongoing training to PAs in addition to initial onboarding training	– Continuous learning and improvement (i.e., obtaining patients experiences and perspectives to direct improvements in various care clinical initiatives) – Interdisciplinary collaboration (i.e., management personnel, staff, patients, and caregivers) – Patient‐centered care (patients actively involved in contributing their experiences and perspective to initiatives related to patient care)
Patient Advisory Council	**Requestors:** – Michigan Hospital Medicine Safety Consortium (HMS) **Referral:** – Organic outreach to hospital departments responsible for care experience and/or Patient Advisory councils	**Intake form (August 12, 2024):** – Service requested: Placement of Patient Advisors on committee or council – Patient Advisor (PA) eligibility: patients with experience of pneumonia, urinary tract infection, sepsis, and/or has intravascular catheter use – PA commitment: 1‐h quarterly meetings for a 2‐year term – Intake meeting (members: HMS) Safety Quality Assurance Coordinator **Intake meeting (August 15, 2024):** – Attendees: PERC's Project Manager – Introduction and Overview of PERC – PERC's Project Manager provided overview of PERC and services offered with focus on qualitative research – Discussion about objectives and goals for project – Discussion about intake form for recruitment of Patient Advisors (eligibility, demographics, specific hospital location experience) – Services needed: Recruitment of Patient Advisors for council – Budget: Not applicable^b^ **Invoice:** Not applicable^b^ **Project approval:** – Funding: Costs covered by PERC through internal funding; HMS pays $50/meeting to patients participating on the council – Invoice: Not applicable^b^	**Project details:** – Recruitment: September 2024 – Plan: September 2024–October 2024 – Continued quarterly meetings **Start date:** September 2024	– 7 PAs recruited as Advisory Council members in 2024 – 3 PAs recruited as Advisory Council members in 2025 – Member engagement maintained from October 2024‐Present **Lessons learned:** – Eligibility requirements limited the recruitment pool, leading to less diversity in demographics of PAs recruited – In response, after pool was exhausted in 2024, another recruitment push for greater diversity was sent in August 2025, as PERC regularly adds new PAs to the program – 3 additional members were recruited in August 2025	– Patient‐centered care (patients actively involved in contributing their experiences and perspective to initiatives related to patient care)

Abbreviation: %FTE, percent full time equivalent.

^a^ Destination: Grand is the name of Henry Ford Health expansion of its $2.2 billion Detroit campus expansion.

^b^ PERC secures internal system funds for quality improvement initiatives, such as PFACs, to cover PERC staffing and other associated costs.

*Step 1: Awareness/Need Recognition*.

PERC leverages various modalities to advertise and create awareness for its services. PERC's services are advertised through its website [30], HFH's intranet (i.e., OneHenry) and daily electronic newsletter (i.e., Morning Post), social media, word of mouth, and championing by previous project collaborators and senior leadership (e.g., sharing resources, promoting PERC). HFH's recently established research and clinical partnership with Michigan State University also provides PERC opportunities for expanded outreach and collaboration.

*Step 2: Intake Process*.

In the early years of PERC (2015–2019), interested teams would contact a PERC team member to make a request for collaboration. As the number of requests began to increase, PERC began developing a formal intake process, consisting of three parts: Intake Form, Intake Meeting, and Invoice.

### 2.1 Intake Form

The Intake Form was created in REDCap (a secure, web‐based software platform for building and managing online surveys) [31, 32] and is available on PERC's website [33]. Those interested in PERC's services use the Intake Form to provide PERC with important information about the project, ensuring that PERC can prepare and strategize before formally meeting with the requestor(s) (see Data S1). Once an Intake Form is submitted by a requestor, PERC's PM receives an automated email notification. The PM reviews the Intake Form and schedules an Intake Meeting.

### 2.2 Intake Meeting

The Intake Meeting exists to help the PERC team understand the requestor's project needs and budget. Within 2 business days of receiving an Intake Form, PERC's PM will schedule a 30‐min virtual Intake Meeting between the requestor(s) and the relevant PERC team members to take place within a 2–3‐week timeframe (sooner if the project is urgent). Meeting invitations are sent as a Microsoft Outlook calendar invitation, with the completed Intake Form attached for reference. Led by the PM the meeting begins with introductions, followed by the requestor's project overview, budget, and desired services. If unsure, PERC helps guide the requestor(s). For first‐time collaborators, PERC reviews its services and fee structure. The meeting also covers participant compensation, whether it will be provided, and if PERC should manage it. While PERC follows PCORI's Compensation Framework [34] and offers guidance on amounts and payment methods, the requestor ultimately decides if and how to compensate participants (i.e., including *amount*—suggested minimum of $50/h, *frequency*—milestone or hourly based, *payment method*—reloadable prepaid card or onetime gift card). Research projects typically include compensation, while quality improvement initiatives often rely on volunteers. For returning requestors, the meeting focuses on agreeing on services based on project needs and budget.

### 2.3 Invoice

After the PM synthesizes all details from the Intake Meeting, an invoice is created, typically offering two to three budget options with varying prices and services. This range helps requestors choose what fits their project needs and budget. PERC is flexible, allowing teams to select services à la carte. Fees are based on an hourly rate, charged as a flat fee, percent full‐time equivalent (%FTE), or both, with rates varying by task (e.g., project management, recruitment) per HFH's Department of Public Health Sciences fee structure [35, 36]. Once the invoice is sent, requestors can discuss and adjust it with the PM to find the best fit.

For requestors solely looking to leverage PERC's PA Program to identify PAs to sit on committees or councils within HFH (or abroad), an invoice is not created. Rather, PERC supports continuous health system learning by using allocated internal funds. Requests to establish Patient Family Advisor Councils (PFACs) not associated with research grants but for HFH learning and improvement are also exempt from invoicing. This model supports operational and clinical division learning across HFH.

*Step 3: Project Approval*.

After the intake process is complete (Intake Form, Intake Meeting, and Invoice, where applicable), PERC waits to receive confirmation that a project is moving forward. If the project has an invoice attached to it, the invoice must be signed by the requestor(s) and returned to PERC's PM before the project can formally move forward.

*Step 4: Project Details and Start Date*.

Once a project is green‐lit, PERC will work with the project team to establish a detailed timeline (including start date), identify key milestones/deliverables that PERC is responsible for, determine preferred communication strategies, and work through payment logistics.

## 3 Discussion

PERC has learned many important lessons pertaining to its SFF. These lessons and related strategies are discussed in depth below.

### 3.1 Creating Buy‐In and Enthusiasm

The value of PE in research, quality improvement, and clinical care is not universally recognized. PERC has, and continues to, spend considerable time obtaining buy‐in for PE from key stakeholders across the health system and within the broader community (e.g., Michigan Health and Hospital Association, Michigan Hospital Medicine Safety Consortium, University of Michigan, Michigan State University, UCLA/VA Center of Excellence on Veteran Resilience and Recovery, Quest Research Institute). Over time, PERC has built strong relationships with well‐respected researchers, clinicians, and members of senior leadership who act as champions for PERC and PE. In doing so, PERC's visibility within the health system and the broader community has grown, resulting in a greater number of requests for collaboration relating to patient‐centered services (which support PE infrastructure).

### 3.2 Tracking of Activities

For years, PERC leveraged its SFF without formal metric tracking. As a result, PERC struggled to quantify its year‐over‐year success and adequately construe the value it was providing to the health system. Thus, from January 2023 onward, PERC began formally tracking all of its SFF activities, including requests for collaboration, work generated (active projects and funds received), presentations delivered (e.g., leadership meetings, research conferences, working groups), and the proportion of PAs serving on placements. For example, in its first year of tracking (i.e., 2023), PERC received 36 total requests for collaboration. Of those 36 requests, 19 were approved for collaboration, with requests for collaboration coming from 10 different departments within HFH, and 5 additional requests from external organizations (e.g., University of Michigan: Seniors using Technology to Engage in Pain Self‐management—PFAC). Additionally, 64% of PAs actively served on a placement from January 2023 to May 2024, with 94 new PAs joining the program in 2023 alone. This number is up to 70% of PAs actively serving on a placement (as of September 2025). However, PERC learned—through a review of its open projects (i.e., those that had intake but were awaiting project approval)—that improvements to its tracking and intake processes were needed. For example, PERC updated its Intake Form to require that requestors applying for grant funding provide the funding notification date, allowing PERC to follow up on this date and learn whether the project will move forward.

### 3.3 Timelines Change

Using the SFF requires PERC to maintain a level of flexibility and adaptability when working with requestors, especially with regards to timelines and budget. Although PERC works with requestors to determine project timelines, factors outside of the requestor or PERC's control often accelerate or delay timelines. For example, receipt of funds or hold‐ups with institutional review board approval can result in a project timeline being pushed back.

### 3.4 Budget Flexibility

As previously mentioned, PERC's SFF provides requestors with flexibility when selecting services and fee structures. Although PERC prefers to budget for services based on %FTE of team members who will be working on each project, PERC understands that projects are often constrained by budgetary limitations. PERC has learned that maintaining flexibility during budget creation and providing the requestors with several potential budgetary/service options is most appropriate.

### 3.5 Projecting the Future

For each request for collaboration received, PERC needs to ensure that it has time to support the proposed collaboration. However, the reality is that not all proposed collaborations come to fruition (e.g., if reliant on grant funding and the funding application is unsuccessful) which creates scheduling challenges. PERC strives to maximize engagement and productivity while avoiding overextension of staff and infrastructure. Thus, learning to predict the likelihood that projects will go forward (e.g., estimating odds a grant may be funded), mapping out all potential and active project timelines, and seeing how each new request for collaboration might overlap with these timelines is extremely important and has helped PERC feel confident in taking on new requests. Similarly, although PERC wants to grow the size of its current team, it is cautious to ensure that the demand for services is consistent and strong enough to support the hiring of additional team members.

### 3.6 Leading by Example

In addition to offering patient‐centered services to support broader patient engagement at HFH and within the broader community through its SFF, PERC is also committed to leading its own research agenda. PERC's researchers seek out grant funding opportunities to lead patient‐engaged research projects of their own, further diversifying its funding. In 2023 alone, PERC successfully secured funding from the Patient Centered Outcomes Research Institute (PCORI) and Blue Cross Blue Shield to lead three projects. Along with helping to support PERC's overall financial sustainability, securing its own research funding allows PERC to showcase the value its staff can provide to future collaborators interested in PE and PERC's patient‐centered services.

## 4 Conclusion

PE in research, quality improvement, and clinical care within most learning health systems is often siloed and disjointed, where those wanting to incorporate PE into their work are left to do so independently. Learning health care systems thrive through practicing continuous learning and improvement, which is integral to PERC's work and its SFF. PERC provides a promising and replicable example by which aspiring and established learning health systems can feasibly sustain and grow PE across research, qualitative improvement, and clinical care through the delivery of à‐la‐carte patient‐centered services.

## Ethics Statement

The authors have nothing to report.

## Consent

The authors have nothing to report.

## Conflicts of Interest

The authors declare no conflicts of interest.

## Supporting information


**Data S1:** lrh270047‐sup‐0001‐Supinfo.pdf.

## References

[1] L. P. Forsythe , K. L. Carman , V. Szydlowski , et al., “Patient Engagement in Research: Early Findings From the Patient‐Centered Outcomes Research Institute,” Health Affairs 38, no. 3 (2019): 359–367. 30830822 10.1377/hlthaff.2018.05067

[2] L. Olsen , D. Aisner , and J. M. McGinnis , “The Learning Healthcare System: Workshop Summary,” 2007. 21452449

[3] J. M. McGinnis , H. V. Fineberg , and V. J. Dzau , “Advancing the Learning Health System,” New England Journal of Medicine 385, no. 1 (2021): 1–5. 34192452 10.1056/NEJMp2103872

[4] C. D. Mullins , L. M. T. Wingate , H. A. Edwards , T. Tofade , and A. Wutoh , “Transitioning From Learning Healthcare Systems to Learning Health Care Communities,” Journal of Comparative Effectiveness Research 7, no. 6 (2018): 603–614. 29478331 10.2217/cer-2017-0105

[5] J. Geissler , B. Ryll , S. L. di Priolo , and M. Uhlenhopp , “Improving Patient Involvement in Medicines Research and Development: A Practical Roadmap,” Therapeutic Innovation & Regulatory Science 51, no. 5 (2017): 612–619. 30231692 10.1177/2168479017706405

[6] L. E. Vat , T. Finlay , T. Jan Schuitmaker‐Warnaar , et al., “Evaluating the “Return on Patient Engagement Initiatives” in Medicines Research and Development: A Literature Review,” Health Expectations 23, no. 1 (2020): 5–18. 31489988 10.1111/hex.12951 PMC6978865

[7] S. Marzban , M. Najafi , A. Agolli , and E. Ashrafi , “Impact of Patient Engagement on Healthcare Quality: A Scoping Review,” Journal of Patient Experience 9 (2022): 23743735221125439. 36134145 10.1177/23743735221125439 PMC9483965

[8] M. Park and T. T. T. Giap , “Patient and Family Engagement as a Potential Approach for Improving Patient Safety: A Systematic Review,” Journal of Advanced Nursing 76, no. 1 (2020): 62–80. 31588602 10.1111/jan.14227

[9] K. L. Carman , P. Dardess , M. Maurer , et al., “Patient and Family Engagement: A Framework for Understanding the Elements and Developing Interventions and Policies,” Health Affairs 32, no. 2 (2013): 223–231. 23381514 10.1377/hlthaff.2012.1133

[10] Patient‐Centered Outcomes Research Institute , “Glossary,” https://www.pcori.org/funding‐opportunities/what‐you‐need‐know‐apply/glossary.

[11] J. Laurance , S. Henderson , P. J. Howitt , et al., “Patient Engagement: Four Case Studies That Highlight the Potential for Improved Health Outcomes and Reduced Costs,” Health Affairs 33, no. 9 (2014): 1627–1634. 25201668 10.1377/hlthaff.2014.0375

[12] A. Coulter and J. Ellins , “Effectiveness of Strategies for Informing, Educating, and Involving Patients,” BMJ 335, no. 7609 (2007): 24–27. 17615222 10.1136/bmj.39246.581169.80 PMC1910640

[13] A. Coulter , “Patient Engagement—What Works?,” Journal of Ambulatory Care Management 35, no. 2 (2012): 80–89. 22415281 10.1097/JAC.0b013e318249e0fd

[14] A. H. Krist , S. T. Tong , R. A. Aycock , and D. R. Longo , “Engaging Patients in Decision‐Making and Behavior Change to Promote Prevention,” Information Services and Use 37, no. 2 (2017): 105–122. PMC6996004 28972524

[15] P. A. Charmel and S. B. Frampton , “Building the Business Case for Patient‐Centered Care,” Healthcare Financial Management 62, no. 3 (2008): 80–85. 19097611

[16] M. J. Armstrong , L. M. Shulman , J. Vandigo , and C. D. Mullins , “Patient Engagement and Shared Decision‐Making: What Do They Look Like in Neurology Practice?,” Neurology: Clinical Practice 6, no. 2 (2016): 190–197. 27104070 10.1212/CPJ.0000000000000240 PMC4828680

[17] J. P. Domecq , G. Prutsky , T. Elraiyah , et al., “Patient Engagement in Research: A Systematic Review,” BMC Health Services Research 14, no. 1 (2014): 1–9. 24568690 10.1186/1472-6963-14-89 PMC3938901

[18] J. Vandigo , E. Oloyede , A. Aly , A. L. Laird , C. E. Cooke , and C. D. Mullins , “Continuous Patient Engagement in Cardiovascular Disease Clinical Comparative Effectiveness Research,” Expert Review of Pharmacoeconomics & Outcomes Research 16, no. 2 (2016): 193–198. 26950206 10.1586/14737167.2016.1163222

[19] H. A. Olden , S. Santarossa , D. Murphy , C. C. Johnson , and K. E. Kippen , “Bridging the Patient Engagement Gap in Research and Quality Improvement Utilizing the Henry Ford Flexible Engagement Model,” Journal of Patient‐Centered Research and Reviews 9, no. 1 (2022): 35–45. 35111881 10.17294/2330-0698.1828 PMC8772608

[20] D. Bloom , J. Beetsch , M. Harker , et al., “The Rules of Engagement: CTTI Recommendations for Successful Collaborations Between Sponsors and Patient Groups Around Clinical Trials,” Therapeutic Innovation & Regulatory Science 52, no. 2 (2018): 206–213. 29714514 10.1177/2168479017720247 PMC5846850

[21] E. M. Perfetto , L. Burke , E. M. Oehrlein , and R. S. Epstein , “Patient‐Focused Drug Development: A New Direction for Collaboration,” Medical Care 53, no. 1 (2015): 9–17. 25494232 10.1097/MLR.0000000000000273

[22] A. Hunter , K. Facey , V. Thomas , et al., “EUPATI Guidance for Patient Involvement in Medicines Research and Development: Health Technology Assessment,” Frontiers in Medicine 5 (2018): 231. 30238004 10.3389/fmed.2018.00231 PMC6136274

[23] I. Klingmann , A. Heckenberg , K. Warner , et al., “EUPATI and Patients in Medicines Research and Development: Guidance for Patient Involvement in Ethical Review of Clinical Trials,” Frontiers in Medicine 5 (2018): 251. 30246010 10.3389/fmed.2018.00251 PMC6137130

[24] K. Warner , W. See , D. Haerry , I. Klingmann , A. Hunter , and M. May , “EUPATI Guidance for Patient Involvement in Medicines Research and Development (R&D); Guidance for Pharmaceutical Industry‐Led Medicines R&D,” Frontiers in Medicine 5 (2018): 270. 30356834 10.3389/fmed.2018.00270 PMC6190844

[25] Team TBTC , M. Elmer , C. Florek , et al., “Amplifying the Voice of the Patient in Clinical Research: Development of Toolkits for Use in Designing and Conducting Patient‐Centered Clinical Studies,” Therapeutic Innovation & Regulatory Science 54, no. 6 (2020): 1489–1500. 32617912 10.1007/s43441-020-00176-6 PMC7704503

[26] K. Deane , L. Delbecque , O. Gorbenko , et al., “Co‐Creation of Patient Engagement Quality Guidance for Medicines Development: An International Multistakeholder Initiative,” BMJ Innovations 5, no. 1 (2019): 43–55. 10.1136/bmjinnov-2018-000317 PMC6792320 31645992

[27] S. Bortoli , “NIHR Guidance on Co‐Producing a Research Project. National Institute for Health Research Southampton: Involve,” 2021.

[28] A. Redding , S. Santarossa , D. Murphy , et al., “A Patient Perspective on Applying Intermittent Fasting in Gynecologic Cancer,” BMC Research Notes 16, no. 1 (2023): 190. 37644560 10.1186/s13104-023-06453-5 PMC10466878

[29] Improvement IfH , “Ask Me 3: Good Questions for Your Good Health,” https://www.ihi.org/resources/tools/ask‐me‐3‐good‐questions‐your‐good‐health.

[30] PERC , “About the Patient Engaged Reserach Center (PERC),” https://www.henryford.com/visitors/perc/about.

[31] P. A. Harris , R. Taylor , R. Thielke , J. Payne , N. Gonzalez , and J. G. Conde , “Research Electronic Data Capture (REDCap)—A Metadata‐Driven Methodology and Workflow Process for Providing Translational Research Informatics Support,” Journal of Biomedical Informatics 42, no. 2 (2009): 377–381. 18929686 10.1016/j.jbi.2008.08.010 PMC2700030

[32] P. A. Harris , R. Taylor , B. L. Minor , et al., “The REDCap Consortium: Building an International Community of Software Platform Partners,” Journal of Biomedical Informatics 95 (2019): 103208. 31078660 10.1016/j.jbi.2019.103208 PMC7254481

[33] PERC , “PERC Services,” https://www.henryford.com/visitors/perc/services.

[34] PCORI , “Financial Compensation of Patients, Caregivers, and Patient/Caregiver Organizations Engaged in Pcori‐Funded Research as Engaged Research Partners,” https://www.pcori.org/sites/default/files/PCORI‐Compensation‐Framework‐for‐Engaged‐Research‐Partners.pdf.

[35] Sciences DoPH , “Fee Schedule,” accessed January 30, 2024, https://onehenry.hfhs.org/departments/publichealthsciences/Pages/Fee‐Schedule.aspx.

[36] Sciences DoPH , “Core Services,” accessed January 30, 2024, https://onehenry.hfhs.org/departments/publichealthsciences/Pages/Core‐Services.aspx.

