# Supplementary material for: The Patient Engaged Research Center's Sustainable Funding Framework: A Path Towards Sustainable Patient Engagement in Care and Research Within a Health System
Source: Learn Health Syst. 2025 Oct 30;10(1):e70047. doi: 10.1002/lrh2.70047 (PMC12812488; doi:10.1002/lrh2.70047)
Supplement: Supplementary file 1 — Data S1: lrh270047‐sup‐0001‐Supinfo.pdf. [file LRH2-10-e70047-s001.pdf]

Patient Engaged Research (PERC) Intake Form

Please complete the survey below.

Thank you!

PERC Intake Form (To obtain PERC services complete the following questionnaire. After the submission of this request form, a PERC Team Member will contact you. Thank you.)

Why are you submitting this intake form?
- ◯ Grant Proposal/LOI
- ◯ Existing Funded Research Project
- ◯ Quality Improvement

Funding Agency Name: ____________________

Due to funder by (date): ____________________

Anticipated date of notice of award: ____________________

Link to funding announcement: ____________________

Project timeline (start-end): ____________________

PERC Budget due by (date): ____________________

Due Date to meet (intake meeting): ____________________

Name: ____________________

Email: ____________________

Department(s): ____________________

What is your Project/Council/Committee called? ____________________

Cost Center/Grant number ____________________

What PERC services are you requesting?

- ☐ Focus Groups
- ☐ One-on-One Interviews
- ☐ PFAC Development
- ☐ PFAC Consult (Present to PFAC group)
- ☐ Committee
- ☐ Survey
- ☐ Recruitment
- ☐ Patient Centered Research Design
- ☐ Knowledge sharing and dissemination
- ☐ Not sure yet

How many focus groups/interviews?

____

How many participants?

____

Select the tasks PERC would lead/assist:

- ☐ Moderator guide
- ☐ Survey development (pre/post)
- ☐ IRB support
- ☐ Recruitment
- ☐ Coordination
- ☐ Facilitation
- ☐ Post-focus group report (summary of notes)
- ☐ Qualitative Analysis
- ☐ Preliminary Analysis Report
- ☐ Transcription Outsource

What is your budget for the PERC service you have selected? (Estimated range)

____

Please provide a detailed description of your request:

Please upload relevant documents here

Participant eligibility requirements:

Please include the names and email addresses of those who should be included in the Intake Meeting:

Who referred you to PERC? How did you hear about our services/program?

Additional Comments:

PERC Acknowledgement

☐ I acknowledge that PERC must be credited in all promotional materials, press releases, presentations, and publications related to this project. Any public mention of the project will include recognition of PERC's role in the collaboration.
